# Prelimbic and Infralimbic Neurons Signal Distinct Aspects of Appetitive Instrumental Behavior

**DOI:** 10.1371/journal.pone.0057575

**Published:** 2013-02-27

**Authors:** Anthony Burgos-Robles, Hector Bravo-Rivera, Gregory J. Quirk

**Affiliations:** Departments of Psychiatry and Anatomy & Neurobiology, University of Puerto Rico School of Medicine, San Juan, Puerto Rico; Radboud University, The Netherlands

## Abstract

It is thought that discrete subregions of the medial prefrontal cortex (mPFC) regulate different aspects of appetitive behavior, however, physiological support for this hypothesis has been lacking. In the present study, we used multichannel single-unit recording to compare the response of neurons in the prelimbic (PL) and infralimbic (IL) subregions of the mPFC, in rats pressing a lever to obtain sucrose pellets on a variable interval schedule of reinforcement (VI-60). Approximately 25% of neurons in both structures exhibited prominent excitatory responses during rewarded, but not unrewarded, lever presses. The time courses of reward responses in PL and IL, however, were markedly different. Most PL neurons exhibited fast and transient responses at the delivery of sucrose pellets, whereas most IL neurons exhibited delayed and prolonged responses associated with the collection of earned sucrose pellets. We further examined the functional significance of reward responses in IL and PL with local pharmacological inactivation. IL inactivation significantly delayed the collection of earned sucrose pellets, whereas PL inactivation produced no discernible effects. These findings support the hypothesis that PL and IL signal distinct aspects of appetitive behavior, and suggest that IL signaling facilitates reward collection.

## Introduction

The medial prefrontal cortex (mPFC) is thought to regulate appetitive behavior, by influencing the striatum [1], [2]. The mPFC consists of anatomically discrete subregions: the prelimbic (PL) and infralimbic (IL) cortices, which are thought to contribute to different aspects of appetitive behavior [3]. Support for different functions of PL and IL comes from studies using reinforcer devaluation in rats well trained to press a lever to obtain sucrose reward. Lesioning PL causes appetitive behavior to become insensitive to devaluation, whereas lesioning IL causes appetitive behavior to maintain its sensitivity to devaluation, despite overtraining [4], [5]. These findings have been interpreted as PL and IL contributing to goal-directed and habitual appetitive behaviors, respectively. Yet, it remains to be elucidated whether PL and IL signal distinct components of the instrumental task (e.g., lever pressing or reward anticipation, delivery, or collection).

Previous neuronal recording studies have revealed correlates of appetitive behavior in the mPFC. Most of these correlates are thought to be related to the anticipation of reward, perhaps due to the predictive nature of the instrumental and spatial-based tasks used [6]–[10]. In addition, most previous studies did not distinguish between PL and IL, leaving in doubt the mechanisms by which these mPFC subregions differentially regulate appetitive behavior.

In the present study, we compared the responses of neurons in PL and IL, in rats trained to press a lever for sucrose pellets on a variable interval schedule of reinforcement. We also used local pharmacological inactivation of PL or IL to determine the functional significance of the firing correlates we observed. We observed distinct correlates in PL and IL, corresponding to sucrose delivery and sucrose collection, respectively. Findings from inactivation experiments suggest that IL, but not PL, facilitates the collection of reward.

## Results

### PL and IL Show Temporally Distinct Responses to Sucrose Reward

Rats were trained to press a lever to obtain sucrose pellets prior to the implantation of recording electrodes. Training occurred over seven consecutive days, during which time the reinforcement schedule was gradually reduced from a continuous fixed ratio (FR-1) to a variable interval with sucrose pellets becoming available every 60 s on average (VI-60). The average lever press rates (presses/min) throughout training were as follows: FR-1a: 1.5, FR-1b: 4.3, FR-1c: 7.2, VI-15a: 11.5, VI-15b: 13.7, VI-15c: 14.8, VI-30a: 14.0, VI-30b: 14.5, VI-30c: 15.1, VI-60a: 14.1, VI-60b: 15.1, and VI-60c: 15.1. Repeated measures analysis of variance (ANOVA) revealed a main effect of training (*F*_(11,297)_ = 51.6, *p*<0.0001), indicating significant acquisition of the task. Bonferroni *post-hoc* tests did not detect significant differences among the three VI-60 sessions (*p*’s>0.28), indicating stable performance of rats on the task.

**Figure 1:**
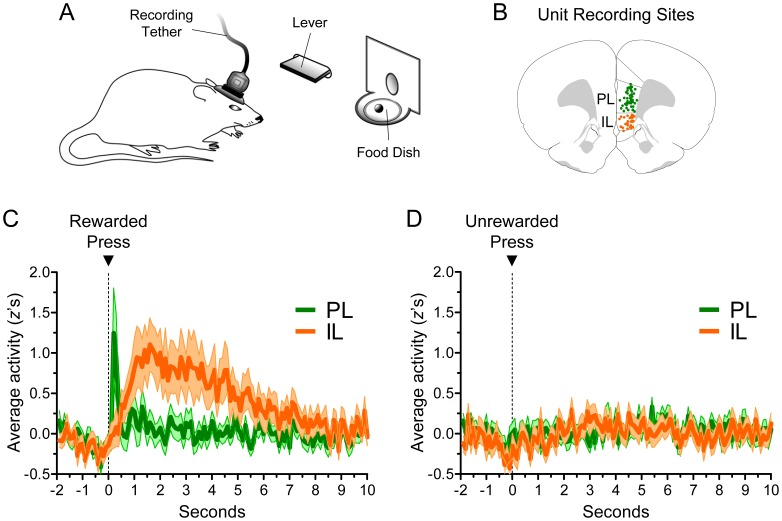
PL and IL exhibited temporally distinct reward responses. ***A,*** Neuronal activity was recorded from PL or IL while rats pressed a lever for sucrose pellets on a variable interval schedule of reinforcement of 60 seconds (VI-60). The lever and food dish were 6 cm apart from each other, and a rewarded press resulted in only one sucrose pellet. ***B,*** Coronal reconstruction of recording sites (bregma +2.80 mm). Dots represent the position of electrode tips, and activity was recorded from a total of 244 neurons from 28 rats (PL: *n* = 134, IL: *n* = 110). ***C,*** Average normalized response of PL and IL during rewarded presses (*all recorded neurons included*). PL exhibited a fast and transient response that lasted for a few hundred milliseconds, whereas IL exhibited a response of longer latency that lasted for several seconds. ***D,*** Neither structure responded during unrewarded presses. [Data is illustrated as *z*-scores, in 100-ms bins. Error in all figures represents SEM.].

Rats were given additional VI-60 training sessions after recording electrodes were implanted, and no significant differences were detected between PL and IL implanted rats (PL rats: 16.1 presses/min, IL rats: 16.3 presses/min; *t*_(26)_ = 0.07, *p* = 0.94). Single-unit activity was recorded from PL and IL while rats pressed on VI-60 (Figure 1A). A total of 244 neurons were recorded from 28 rats (PL neurons: *n* = 134; IL neurons: *n* = 110). A coronal reconstruction of the recording sites is shown in Figure 1B.

Both PL and IL exhibited prominent excitatory responses during rewarded lever presses, however, the time course of their responses was strikingly different. Figure 1C shows the average normalized activity for all neurons recorded from PL and IL during rewarded lever presses. Notice that while the average PL response was fast and transient, the average IL response was delayed and prolonged. The PL response had a latency of ∼0.1 s, and lasted no longer than 0.3 s. In contrast, the IL response ramped up slowly, peaked at ∼1 s after the lever press, and slowly returned to baseline after several seconds. Two-way ANOVA revealed main effects of structure (*F*_(1,242)_ = 5.43, *p* = 0.021), time (*F*_(119,28798)_ = 4.08, *p*<0.001), and interaction (*F*_(119,28798)_ = 2.93, *p*<0.001). The different response profiles of PL and IL support the idea that these mPFC subregions signal different aspects of appetitive behavior.

We next examined neuronal responses during unrewarded lever presses to distinguish whether excitatory responses in PL and IL were related to either the delivery of reward or the lever press itself. Data for unrewarded presses were obtained for a subset of neurons (PL: 108, IL: 79). Neither PL nor IL showed excitatory responses during unrewarded lever presses. As shown in Figure 1D, the only correlate exhibited by PL and IL during unrewarded presses was a small decrease in activity just prior to lever pressing. This anticipatory inhibition was also present during rewarded presses (*see* Figure 1C). Two-way ANOVA showed no significant differences between structures during unrewarded presses (*F*_(1,182)_ = 0.32, *p* = 0.37), but showed a significant effect of time caused by the anticipatory inhibition (*F*_(119,21658)_ = 1.36, *p* = 0.006). Thus, excitatory responses in PL and IL were not related to lever presses, but appear to be related to aspects of the reward. The fast latency of PL responses suggests that they were related to the delivery of reward, whereas the delayed latency of IL responses suggests that they were related to events that followed pellet delivery.

### Transiently-responsive Neurons Predominate in PL, whereas Prolonged-responsive Neurons Predominate in IL

**Figure 2:**
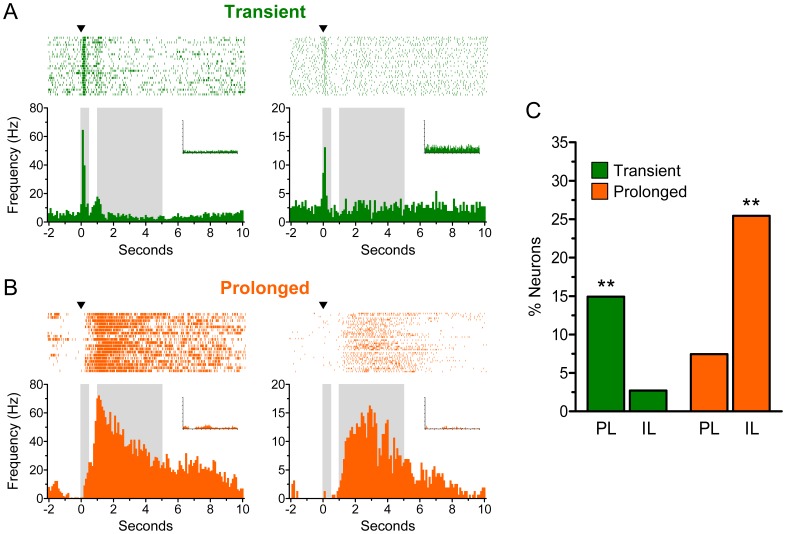
Transiently-responsive neurons predominated in PL, whereas prolonged-responsive neurons predominated in IL. ***A-B,*** Single-neuron examples of transient and prolonged excitatory responses during rewarded lever presses. Insets show the activity of these neurons during unrewarded presses, and gray boxes represent the temporal windows used to quantify the proportion of neurons showing each type of response. ***C,*** Percentage of neurons per structure that showed either transient or prolonged excitation during rewarded lever presses. PL exhibited significantly more transiently-responsive neurons than IL, whereas IL exhibited significantly more prolonged-responsive neurons than PL (Χ^2^ tests: **p<0.01).

To further explore differences between PL and IL, we quantified the proportion of neurons showing either transient or prolonged excitation in each structure. Neurons were classified as transiently-responsive if they exhibited at least 1 significant excitatory bin (100-ms duration) within 500 ms of a rewarded press (*representative neurons are shown in* Figure 2A). In contrast, neurons were classified as prolonged-responsive if they exhibited 5 or more significant excitatory bins within 1 and 5 s of a rewarded press (*representative neurons are shown in* Figure 2B). A significance level of *p*<0.001 (i.e., *z*-scores>3.29; *see Materials and Methods for details*) was used to detect significant excitatory bins.

Although both types of neurons were present in both structures, transiently-responsive neurons predominated in PL whereas prolonged-responsive neurons predominated in IL. As shown in Figure 2C, the proportion of neurons showing transient excitation was significantly higher in PL than IL (PL: 20/134, 15%; IL: 3/110, 3%; *X* ^2^ = 8.84, *p* = 0.003). In contrast, the proportion of neurons showing prolonged excitation was significantly higher in IL than PL (IL: 28/110, 25%; PL: 10/134, 7%; *X* ^2^ = 10.8, *p* = 0.001). Using similar criteria (*see Material and Methods for details*), we also detected a small number of inhibitory-responsive neurons. Only 6 PL neurons (4%) and 8 IL neurons (7%) exhibited significant inhibition during rewarded lever presses, which was prolonged in the majority of the cases. There were no statistically significant differences between PL and IL in the proportion of inhibitory-responsive neurons (*Χ^2^* = 0.37, *p* = 0.54). Therefore, the predominant response in PL was transient excitation, whereas the predominant response in IL was prolonged excitation.

### The Latency of IL Responses Resembles the Latency of Reward Collection

**Figure 3:**
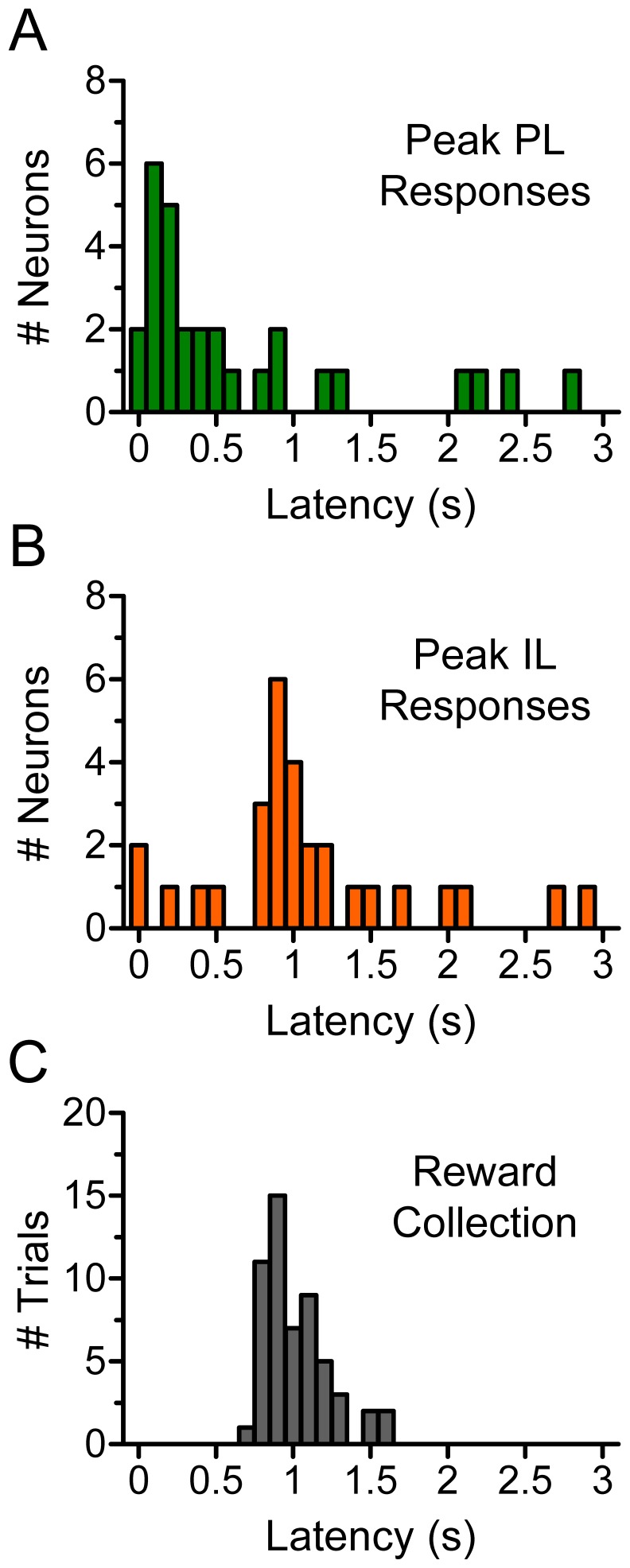
Latencies for PL, IL, and reward collection. The distribution of PL latencies peaked at ∼0.1 s after rewarded lever presses were achieved (*A*), whereas the distribution of IL latencies peaked at ∼1 s (*B*). Similar to IL, the distribution of latencies for reward collection peaked at ∼1 s after rewarded presses were achieved (*C*).

To test the possibility that IL signaling was associated with reward collection, we compared the latency of IL (and PL) responses with the latency for reward collection from a group of unimplanted rats videotaped at 30 frames per second, using an angle of view that allowed us to accurately determine when rats placed the sucrose pellets in their mouths. As shown in Figure 3, the distribution of latencies for IL responses and reward collection both peaked at ∼1 s after lever pressing. In contrast, the distribution of PL latencies peaked at ∼0.1 s after lever pressing. Thus, the remarkable similarity between the latencies of IL (but not PL) and reward collection is consistent with the hypothesis that IL responses were associated with the collection of earned sucrose pellets.

### Inactivation of IL, but not PL, Significantly Delayed Reward Collection

If IL activity truly contributes to reward collection, then pharmacological inactivation of IL should alter the latency of reward collection. We therefore inactivated either IL or PL by locally infusing the GABA_A_ receptor agonist muscimol, at the same dose we previously used to impair the expression of other IL- and PL-dependent memories [11]. One-way ANOVA showed no significant group differences prior to drug infusion (PL-Sal: 15.8±2.0 presses/min, PL-Mus: 16.5±0.8 presses/min, IL-Sal: 14.5±0.9 presses/min, IL-Mus: 14.8±1.0 presses/min; *F*_(3,18)_ = 0.45, *p* = 0.72).

**Figure 4:**
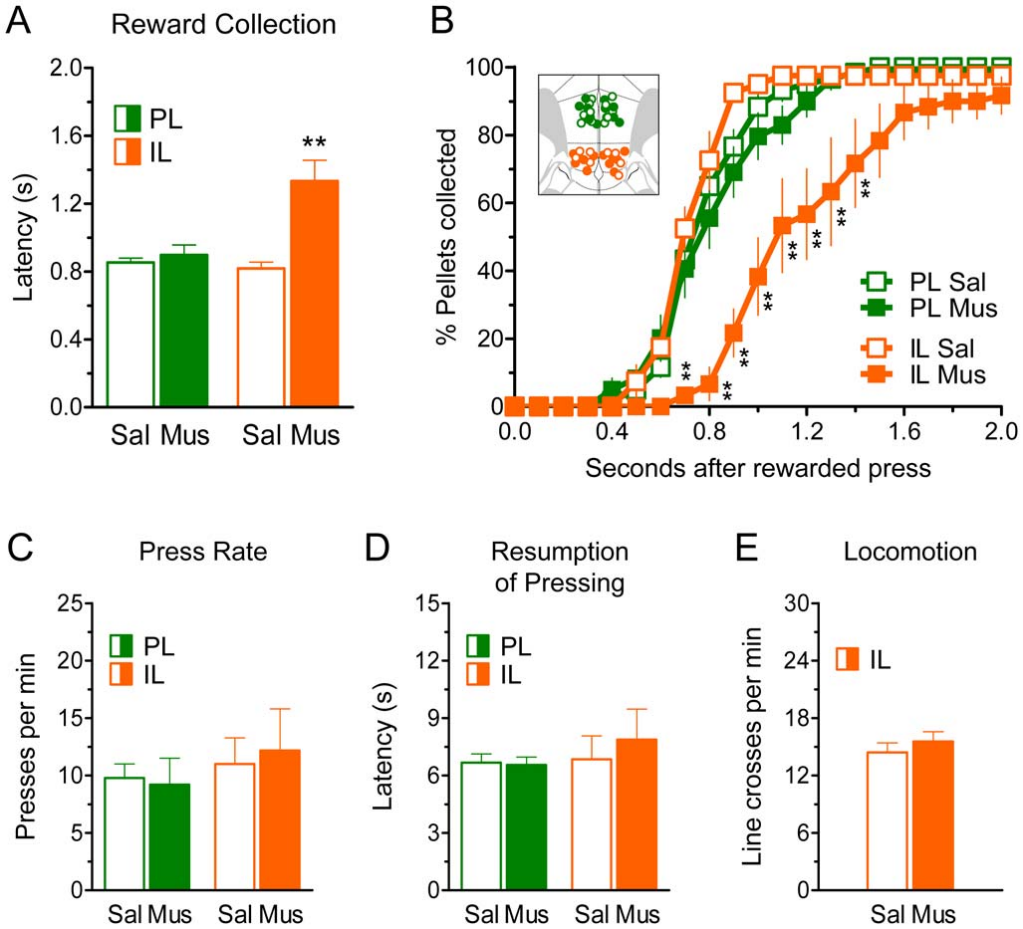
Inactivation of IL, but not PL, altered the time course of reward collection. Either the GABA_A_ agonist muscimol (*Mus*) or saline vehicle (*Sal*) was bilaterally infused into IL or PL 30 min prior to testing animals on VI-60. ***A–B,*** Inactivation of IL, but not PL, significantly delayed the collection of earned pellets (**p<0.01, compared to all other groups). ***C–E,*** Other behavioral measures were unaffected by IL or PL inactivation, including the rate of lever pressing (*C*), latency for the resumption of lever pressing after collecting the earned pellets (*D*), or general locomotion in an open-field test (*E*). [Inset in *B* shows the location of injector tips. PL-Sal: *n* = 6, PL-Mus: *n* = 6, IL-Sal: *n* = 4, IL-Mus: *n* = 6].

As shown in Figure 4A, inactivation of IL, but not PL, significantly increased (>60%) the latency of reward collection. The average collection latencies for the PL-Sal, PL-Mus, IL-Sal, and IL-Mus groups were 853 ms, 898 ms, 819 ms, and 1,334 ms, respectively. One-way ANOVA revealed significant group differences (*F*_(3,18)_ = 10.1, *p*<0.001), and Bonferroni *post-hocs* confirmed that the collection latencies in the IL-Mus group were significantly higher than in all other groups (all *p*’s <0.01). This increase in collection latency is also evident in Figure 4B, which shows the cumulative percentage of pellets collected as a function of time, relative to lever presses (see *Materials and Methods* for details on the generation of these curves). Notice that reward collection in the IL-Mus group was significantly shifted to the right. Two-way ANOVA revealed main effects of treatment (*F*_(3,18)_ = 11.1, *p*<0.001), time (*F*_(20,360)_ = 351.5, *p*<0.001), and interaction (*F*_(60,360)_ = 5.72, *p*<0.001). *Post-hoc* tests confirmed that reward collection in the IL-Mus group was significantly delayed relative to all other groups (all *p*’s <0.01, at all time-bins from 0.7 s to 1.4 s). Also notice in Figure 4B that while reward collection was delayed, IL-Mus rats eventually collected most of the earned pellets (*see* time-bin 2.0 s). *Post-hoc* tests did not detect significant group differences in the percentage of pellets collected at time-bin 2.0 s (all *p*’s>0.26). Thus, appetitive signaling in IL, but not PL, appears to facilitate the collection of reward.

Other behavioral measures were unaffected by IL or PL inactivation. This included the rate of lever pressing (*see* Figure 4C; *F*_(3,18)_ = 0.28, *p* = 0.84), the latency to resume lever pressing after pellet collection (*see* Figure 4D; *F*_(3,18)_ = 0.38, *p* = 0.77), and general locomotion in an open field test (*see* Figure 4E; *t*_(8)_ = 0.76, *p* = 0.47). Collectively, these findings indicate that IL, but not PL, is an important modulator of reward collection.

## Discussion

Although it is generally acknowledged that the mPFC plays a key role in modulating appetitive behaviors, the individual contributions of the PL and IL subregions have not been emphasized. We observed distinct physiological responses in PL and IL capable of differentially influencing appetitive behavior. During our instrumental appetitive task, PL neurons exhibited fast and transient excitatory responses that were associated with the delivery of reward, whereas IL neurons exhibited delayed and prolonged excitatory responses that were associated with reward collection.

PL showed prominent reward responses, yet inactivation of this structure had no observable effects on appetitive behavior in this task. It is important to note that our rats were well trained prior to PL inactivation, and previous studies have shown that blockade of glutamatergic or dopaminergic receptors in PL impairs the initial acquisition of instrumental appetitive behavior [12]. Furthermore, initial acquisition of instrumental behavior increases the level of many activity-dependent and plasticity-related molecular markers in PL [13], [14]. Thus, PL may be more important for the initial acquisition of instrumental responses, rather than their subsequent expression. Nevertheless, PL inactivation in well-trained animals has been shown to reduce cue-evoked reward-seeking behavior [15], [16], suggesting that PL signaling after initial training is still important to facilitate appetitive behavior when reward is predicted by environmentally salient cues.

In contrast to PL, neuronal responses in IL were associated with the collection of reward. Furthermore, IL inactivation significantly delayed (but did not prevent) the collection of reward. This is consistent with a recent study showing that inactivation of IL increases the latency for reward collection in a five-choice serial reaction time task [17]. In addition, previous studies using reinforcer devaluation have determined that IL activity promotes habitual appetitive behavior [4], [18]. Thus, it is possible that the IL activity we observed contributes to the habitual nature of appetitive behavior. A potential output of IL that could modulate reward collection is the lateral hypothalamus (LH), which receives robust monosynaptic inputs from IL and is essential for promoting eating [19], [20]. Another potential output of IL for facilitating appetitive behavior is the central nucleus of the amygdala (CeA), which receives dense inputs from IL and also projects to LH [19], [21], [22].

Projections from IL to CeA have also been implicated in the regulation of conditioned fear [23], [24]. IL neurons are activated by conditioned fear stimuli that have been extinguished [25]–[27], and inactivation of IL prevents extinction of conditioned fear [11], [28], suggesting that IL contributes a safety signal that inhibits fear expression. Our present finding that IL also facilitates reward collection suggests that IL may coordinate fear and appetitive behaviors, for example under conditions in which food collection occurs in the face of predatory threats [29].

## Materials and Methods

### Subjects

All procedures were approved by the Institutional Animal Care and Use Committee of the University of Puerto Rico School of Medicine, in compliance with the PHS Policy on Humane Care and Use of Laboratory Animals (Public Law 99–158). Adult male Sprague-Dawley rats weighting ∼300 g were housed individually, and maintained on a 12 hr light/dark cycle. Rats had free access to water, but food was restricted to 18 g/d of standard laboratory rat chow until rats reached 85% of their free-feeding weight.

### Lever-Press Training

Rats were trained to press a lever to obtain sucrose pellets (45-mg, Bio-Serv). Lever-press training was conducted during light/day hours, and it occurred in a standard operant chamber (30 w×25 d×30 h cm) that was controlled by commercial software (Graphic State, Coulbourn Instruments). The response lever was freely available, and it was positioned ∼6 cm apart from the food tray on the same wall of the chamber. The food magazine was positioned on the outside of the chamber, and it was programmed to drop a single sucrose pellet 20 ms after a rewarded lever press was achieved (20 ms was the maximum sampling rate at which our behavioral setup operated).

Training occurred over seven consecutive days, during which the reinforcement schedule was gradually reduced from a continuous fixed ratio (FR-1) to a variable interval schedule with sucrose pellets becoming available every 60 s on average (VI-60). Each training session lasted 30 min, and in total rats underwent 3 FR-1 sessions, 3 VI-15 session, 3 VI-30 sessions, and 3 VI-60 sessions. Rats pressing less than 10 times per minute by the end of training were not subjected to further experimentation.

### Multichannel Single-Unit Recording

After learning the instrumental task, a total of 28 rats were chronically implanted with movable electrodes targeting either PL or IL. Electrodes consisted of 16 single nickel-chromium microwires (22 µm in diameter; Stablohm 675; California Fine Wire) that were attached to a 10×2 MillMax connector [30], [31]. The tip of each wire was gold plated by applying a 1-µA current for several seconds while the wires were submerged in a gold solution. Gold electroplating reduced the impedance of the wires to a range of 250–350 kΩ. Electrodes were implanted into the mPFC using the following stereotaxic coordinates: 2.9 mm anterior, 0.5–0.8 mm lateral, and 3.5 mm (PL) or 5.0 mm (IL) ventral from bregma [32]. Buprenorphine hydrochloride (Buprenex, i.m., 0.02 mg/kg) was administered to relieve post-operative pain, and rats were allowed 7 d to recover from surgery. Additional lever-press training was given after rats recovered from surgeries to get stable lever press rates.

Extracellular waveforms exceeding a voltage threshold were amplified and digitized at 40 kHz, using a commercial data acquisition package (Plexon Inc). Electrodes were advanced in steps of 20–40 µm until well-isolated units were encountered. Activity from stable units was recorded over 20 min, while rats performed the instrumental task on VI-60. Lever presses (both rewarded and unrewarded) were time-stamped and stored along with the single-unit data. Waveforms were then sorted off-line, using three-dimensional plots of peak and valley voltages and principal component analysis (Offline Sorter, Plexon Inc). Auto-correlograms and cross-correlograms generated from spike trains of simultaneously recorded units were inspected to avoid counting the same cell twice.

Peri-event time histograms were generated with 100-ms bins to examine the response of neurons during both rewarded and unrewarded lever presses (Neuroexplorer, NEX Technologies). Because many unrewarded presses were flanked by rewarded presses, only a subset of unrewarded events (at least 20 per rat) that were not closely flanked by rewarded events (±15 s) was taken into account for analysis. The response of individual neurons was normalized to the baseline period of −5 s to −2 s prior to lever pressing, using a *z*-score transformation. Average peri-event time histograms were generated for PL and IL, and differences between structures were examined using a two-way analysis of variance (ANOVA) with *structure* and *time* as independent factors.

Neurons were categorized according to the type of response they exhibited: (*a*) transient excitation, (*b*) transient inhibition, (*c*) prolonged excitation, or (*d*) prolonged inhibition. Transiently-responsive neurons showed at least one significant bin of 100-ms duration within the first 500 ms after lever pressing, whereas prolonged-responsive neurons showed 5 or more significant bins of 100-ms duration between 1 s and 5 s following a lever press. A significance level of *p*<0.001 was used to detect excitatory bins (i.e., *z*-scores>3.29), and a significance level of *p*<0.01 was used to detect inhibitory bins (i.e., *z*-scores<−2.58). The proportion of neurons showing each type of response was calculated for PL and IL, and structural differences were tested using Chi-square (*X* ^2^).

### Behavioral Analyses

Behavior was examined by an experimenter blind to treatment, from digital videos acquired at 30 frames per second. The following two latencies were measured: (*a*) the latency for sucrose pellet collection following a rewarded press, and (*b*) the latency for the resumption of lever pressing following pellet collection. The latencies for pellet collection were used to generate pellet collection curves as functions of time, using a binary code. During a rewarded event, scores of ‘0’ were assigned to each 100-ms bin, between the occurrence of the lever press and the collection of the pellet from the dish (i.e., pellet in mouth). Then, from the time of pellet collection, scores of ‘1’ were assigned to each following 100-ms bin. Averaging across many rewarded events yielded cumulative collection curves for each rat. A two-way ANOVA with *treatment* and *time* as independent factors was used to compare the collection curves across treatment groups. Bonferroni *post-hoc* tests were used to detect group differences in time-bin values.

### Pharmacological Inactivation

Rats were bilaterally implanted with 26-gauge stainless-steel guide cannulas (Plastics One) in either PL or IL. PL cannulas were double-barreled, and were positioned at stereotaxic coordinates 2.9 mm anterior, 0.5 mm lateral, and 3.0 mm ventral from bregma [32]. IL cannulas were single-barreled (thus, two per rat), and were positioned at an angle of 30° towards the midline, thus preventing backflow to PL [11]. Stereotaxic coordinates for IL cannulas were 2.9 mm anterior, 3.1 mm lateral, and 4.0 mm ventral from bregma [32].

One day prior to drug infusion, obturators were removed and replaced with 33-gauge stainless-steel injectors that extended 1 mm beyond the tip of the guide cannulas. During the day of drug infusion, obturators were replaced again with injectors connected to 10-µL syringes (Hamilton Co), using PE-20 polyethylene tubing. Drugs were infused 30 min prior to testing the rats in the instrumental task (on VI-60), at a rate of 0.2 µL per min, using an infusion pump (Harvard Apparatus). After infusing the drugs, injectors were left in place for an additional minute to allow drugs to diffuse. Injectors were then removed, obturators were replaced, and rats were returned to their homecages. Rats in the control groups were infused with 0.9% sterile saline, and rats in the inactivation groups were infused with the GABA_A_ (γ-amino butyric acid type A) receptor agonist muscimol at a dose of 0.11 nmol (12.5 ng in 0.2 µL, per hemisphere). Prior studies from our laboratory have demonstrated that this dose of muscimol is sufficient to impair the expression of PL and IL-dependent behaviors [11].

### Open-Field Test

An open-field test was conducted prior to euthanizing the rats to assess the effects of pharmacological inactivation on general locomotion. The open field had dimensions of 91.5 w×91.5 d×61 h cm, and lines were drawn on the floor of the apparatus to form a 5×5 grid. Rats were infused with either saline or muscimol (at the dose stated above) 30 min prior to a 10-min open-field session. Open-field sessions were videotaped, and locomotion was quantified by an experimenter blind to treatment, by counting the total number of line crosses. Group differences were examined using unpaired T-tests.

### Histology

Rats were euthanized with a high dose of pentobarbital (150 mg/kg, i.p.). Recording sites were marked by passing a 25-µA current for 20 s. Rats were transcardially perfused with 0.9% saline and 10% buffered formalin. Brains were removed and fixed in a formalin solution containing 30% sucrose and 6% ferrocyanide, which produced a green iron deposit around the microlesion. Coronal sections were cut at 40 µm, mounted on slides, and stained for Nissl bodies. Coronal sections were examined using a light microscope with digital image processing capability (Nikon Instruments Inc). Unit recording sites were mapped onto coronal drawings adapted from a stereotaxic brain atlas [33]. The sites of drug infusion were also reconstructed from Nissl-stained coronal sections.
